# Haemorrhage from the Ear

**Published:** 1858-01

**Authors:** H. P. Strong


Beloit, Dec. 2d, 1857.

Dr. Hunt,—Since writing you a few days since, another case of hæmorrhage from the ear has occurred under my observation.

J. G., while riding along a business street was thrown suddenly from his wagon by another team which was running. He fell with great force, striking upon the left side of the head. He remained in the position assumed by the fall, some minutes, when taken up by friends. When raised up the blood streamed from the left ear quite profusely, and continued for some twenty or thirty minutes

Partial reäction took place; sat up; semiconscious; vomited some, and was taken home, some mile distant. After getting home he grew comatose until stertorious breathing was seen, and remained in this condition forty-eight hours, when death took place.

Fracture of the base of the skull was "*diagnosticated*," (according to the Boston Med. Jour.,) by a physician, (not myself,) and an operation proposed, viz., "cutting down upon the occipital bone to *find* depression for the application of the trephine," and was performed with no perceptible result.

*Post-mortem* revealed a fracture of the petrous portion of the temporal bone, and an extravasation of blood at the base extending to the thalamus-opticus, to the amount of half a pound.

Yours, H. P. Strong.

---

*Improvement in Methods of rendering Medicinal Preparations pleasing to the eye and to the taste, and agreeable to use.* By Frederick Stearns, Pharmaceutist, Detroit, Mich.

The above is the title of a paper read before the American Pharmaceutical Association, at its sixth annual meeting, by our former townsman, Mr. Stearns, now of Detroit. All practitioners have experienced and continue to experience, great annoyance, from the unwillingness of many of their patients to take disagreeable remedies, and we are often deceived by our patients throwing away their medicines, thereby exposing the doctor to perplexity in not being able to understand why his remedies produce no effect; or, what is equally common, leading him to exult over a rapid and gratifying cure, when the patient and friends know that nature alone is entitled to the credit. Under the latter circumstance, especially the physician, is apt to lose some of the reputation for omniscience which it is so essential to establish and maintain.

In looking over the paper by Mr. Stearns, we notice a number of exceedingly useful suggestions in this department; our personal knowledge of the author makes us feel an interest in his welfare; he is now working with the right spirit and in the right way, and we predict that he must succeed. F.

---

*Medical Lexicon. A Dictionary of Medical Science.* By ROBLEY DUNGLISON, M. D., LL. D., Professor of the Institutes of Medicine, etc., in the Jefferson Medical College of Philadelphia. Revised and very greatly enlarged. Philadelphia: Blanchard & Lea. 1857.

This work, the appearance of the fifteenth edition of which, it has become our duty and pleasure to announce, is perhaps the most stupendous monument of labor and erudition in medical literature. One would hardly suppose after constant use of the preceding editions, where we have never failed to find a sufficiently full explanation of every medical term, that in this edition "*about six thousand subjects and terms have been added*," with a careful revision and correction of the entire work. It is only necessary to announce the advent of this edition to make it occupy the place of the preceding one, on the table of every medical man, as it is without doubt the best and most comprehensive work of the kind which has ever appeared.

Notwithstanding the increased size, the price still remains only $4.00.

F.

---

*A new Journal at Louisville, Ky.*—We see by the Nashville Journal, that Dr. D. W. Yandell contemplates the founding of a new journal of medicine in that city, to supply the place of the Louisville Review, which was transplanted to Philadelphia and associated with the Medical Examiner. We have the pleasure of a personal acquaintance with Dr. Yandell, and know him to be admirably calculated for the task which he has in view. The readers of the Western Medical Journal, will recollect the initials D. W. Y., which appeared in that Journal, at the foot of articles which contributed greatly to the interest of its pages. F.

---

*American Veterinary Journal.*—We notice that the cover of our friend the American Veterinary Journal, is divested of that remarkable series of equine representations which formerly adorned it, and has assumed a less striking, but more modest and engaging aspect. Though we have not devoted especial attention to this branch, we have often read with pleasure, articles which have appeared in its pages, and those interested in such matters, are to be congratulated that veterinary science has so able an advocate in this country. F.

---

*The North American Medical and Surgical Journal.*—The long and now inappropriate title of this excellent Journal, will be changed in the succeeding volume, and the January number will appear under the title of the Chicago Medical Journal. Chicago can now hardly be called the North-West, as it was when this Journal was instituted; this change in the name therefore, seems to be called for. The Chicago Journal we have no doubt, will well sustain the high stand which it has taken as The North-Western·

F.

---

*Geo. R. Gliddon, Esq.*, formerly United States Consul at Cairo, Egypt, and distinguished for his contributions to antiquarian science, died at Panama, on the 11th Nov. Mr. Gliddon had been on a visit to Honduras, as agent of the Honduras Interoceanic Railway Company, and was on his way to the United States when overtaken by death.

Mr. Gliddon was the principal contributor to "Types of Mankind," and "Indigenous Races of Men," and a prominent advocate of the views of the new school of ethnology.—*Medical & Surgical Reporter.*

